# Non-Permissive Parvovirus B19 Infection: A Reservoir and Questionable Safety Concern in Mesenchymal Stem Cells

**DOI:** 10.3390/ijms24098204

**Published:** 2023-05-03

**Authors:** Gloria Bua, Pasquale Marrazzo, Elisabetta Manaresi, Chiara Gamberini, Laura Bonsi, Francesco Alviano, Giorgio Gallinella

**Affiliations:** 1 Department of Pharmacy and Biotechnology, University of Bologna, 40138 Bologna, Italy; gloria.bua2@unibo.it (G.B.); elisabetta.manaresi@unibo.it (E.M.); giorgio.gallinella@unibo.it (G.G.); 2 Department of Medical and Surgical Sciences, University of Bologna, 40138 Bologna, Italy; pasquale.marrazzo2@unibo.it (P.M.); chiara.gamberini15@unibo.it (C.G.); laura.bonsi@unibo.it (L.B.); 3 Department of Biomedical and Neuromotor Sciences, University of Bologna, 40126 Bologna, Italy

**Keywords:** parvovirus B19, mesenchymal stem cells, persistent infection, bone marrow, placenta

## Abstract

Mesenchymal stromal/stem cells (MSCs) are multipotent cells with differentiation, immunoregulatory and regenerative properties. Because of these features, they represent an attractive tool for regenerative medicine and cell-based therapy. However, MSCs may act as a reservoir of persistent viruses increasing the risk of failure of MSCs-based therapies and of viral transmission, especially in immunocompromised patients. Parvovirus B19V (B19V) is a common human pathogen that infects bone marrow erythroid progenitor cells, leading to transient or persistent anemia. Characteristics of B19V include the ability to cross the placenta, infecting the fetus, and to persist in several tissues. We thus isolated MSCs from bone marrow (BM-MSCs) and fetal membrane (FM-MSCs) to investigate their permissiveness to B19V infection. The results suggest that both BM- and FM- MSCs can be infected by B19V and, while not able to support viral replication, allow persistence over time in the infected cultures. Future studies are needed to understand the potential role of MSCs in B19V transmission and the conditions that can favor a potential reactivation of the virus.

## 1. Introduction

Mesenchymal stromal/stem cells (MSCs) constitute a heterogeneous population of multipotent precursor cells with high in vitro expansion and self-renewal capacity. Their typical functions are the ability to differentiate into multiple mesenchymal lineages, including osteocytes, chondrocytes, adipocytes and endothelial-like cells, and to migrate to sites of tissue injury and inflammation [1]. Because of these features, together with their immunoregulatory and regenerative properties, they represent an attractive tool for tissue engineering applications and cell-based therapy [2]. MSCs have been isolated from many sources, including adult tissues, as well as several fetal and perinatal tissues [3,4]. Bone marrow (BM) is the most common source of clinical application, and the properties of BM-derived MSCs (BM-MSCs) have been extensively investigated. However, they constitute a small percentage of the total number of BM-populating cells, and their isolation requires invasive procedures. Searching for a valid alternative, the term placenta represents an easily available and high-yielding source of stem cells [5]. Fetal membranes-derived MSCs (FM-MSCs) have been shown to possess MSC minimal criteria, high plasticity and high differentiation potential [6,7]. In addition, their low immunogenicity and fetal–maternal tolerance make them promising in therapeutic applications without immunologic rejection [8]. Although clinical applications of MSCs transplantation have shown promising achievements, the main drawbacks are the in vivo efficacy and safety profile. One of the major concerns is the mostly hidden relationship between viruses and MSCs. The outcomes of viral entry into MSCs could result in cell death, the alteration of cell properties that could impair their functionality and therapeutic efficacy, the transmission of viral agents, and persistent infection with potential reactivation in the host patient.

Parvovirus B19 (B19V) is a human pathogenic virus with a specific tropism for bone marrow erythroid progenitor cells (EPC) [9,10,11]. Although B19V infection is usually asymptomatic or mild in healthy individuals, severe outcomes are possible according to the physiological and immune status of the infected person [12]. Productive infection of EPC exerts a cytotoxic effect and causes the blockage of erythropoiesis, which can manifest as acute aplastic crises in subjects with high red cell turnover, or chronic pure red cell aplasia in immunodeficient patients, including bone marrow or solid organ transplant recipients [13]. Vertical transmission may also occur from mother to fetus, increasing the risk of serious complications such as anemia, fetal death and non-immunologic hydrops fetalis [14]. Knowledge regarding the mechanism by which the virus breaches the placental barrier and the effects of B19V infection in cells of the maternal–fetal interface is still lacking. Following a primary infection, the virus can be detected at high frequency, either silent or associated with low-grade replication, in almost all solid tissues and organs [15,16,17]. This close association is assumed to persist throughout life, posing questions about the biological role of persistent DNA, as to whether re-activation can take place and the implications of the latent state for the cellular environment, with a special focus on bone marrow. The evaluation of the cell types that could be infected by B19V and the potential effects of the resulting virus–cell interaction is thus particularly important, both in understanding the pathogenic role of B19V and assessing safety issues concerning biological material for cell therapy, such as MSCs.

To this end, in the present study, we investigated the susceptibility and permissiveness of BM-MSCs and FM-MSCs to B19V infection.

## 2. Results

### 2.1. Characterization of Isolated MSCs

**Figure 1:**
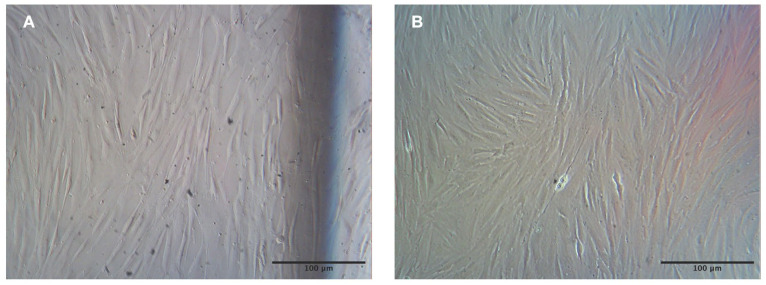
Light microscopy images of expanded BM-MSCs (**A**) and FM-MSCs (**B**).

BM- and FM-MSCs were isolated as previously reported [7]. Cells met the minimal criteria for defining MSCs recommended by the Mesenchymal and Tissue Stem Cell Committee of the International Society for Cellular Therapy [1]. Fibroblast-like morphology and adherence to plastic were first confirmed by light microscopy (Figure 1).

**Figure 2:**
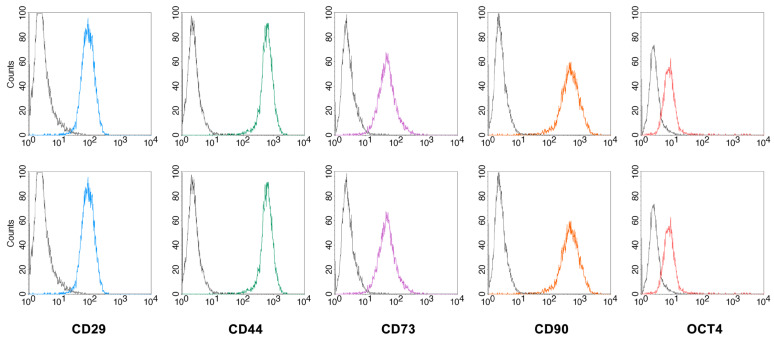
Immunophenotype of BM-MSCs (**top**) and FM-MSCs (**bottom**). Selected mesenchymal markers were analyzed by flow cytometry. Black line: control; colored line: specific antibody.

To further characterize the isolated MSCs, the expression of specific surface markers was assessed by flow cytometry (Figure 2). The cells expressed the surface antigens CD29, CD44, CD73 and CD90 (100% positive cells for all markers), indicating that these cells were of the mesenchymal lineage. The intracellular expression of OCT4, a regulatory transcription factor involved in the maintaining of stem cell renewal capacity and the undifferentiated state of embryonic and adult stem cells, was also confirmed (positive cells > 80%).

### 2.2. Expression of Viral Receptors on MSCs

**Figure 3:**
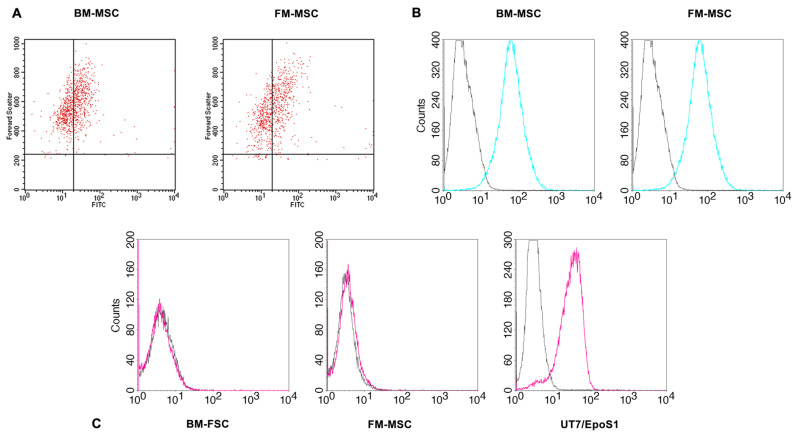
Expression of B19V receptors in BM-MSCs and FM-MSCs determined by flow cytometry. (**A**) Globoside expression; events located in the up/left quadrant of dot-plot graphs represent positive cells. (**B**) α_5_β_1_ integrin expression; black line: control; colored line: specific antibody. (**C**) Expression of VP1u receptor; black line: control; colored line: specific antibody A truncated form of the B19V-VP1u protein with a FLAG tag was used for detecting the unknown VP1uR. The functionality of the VP1 construct was confirmed in UT7/EpoS1 cells that are known to express the receptor [23].

To determine whether the MSCs were susceptible to B19V infection, the expression of B19V receptors was investigated. B19V requires multiple steps to enter into cells and achieve a productive replicative cycle. Globoside (Gb4) was historically identified as the primary B19V receptor that mediates the interaction with the cell surface [18,19]. Virus uptake, however, is not always possible even in the presence of Gb4, indicating that interaction with co-receptors may be critical. α_5_β_1_ integrin was initially proposed as a co-receptor [20], and recently, new evidence has emerged on the role of the VP1u region that specifically binds a not-yet characterized receptor, named VP1uR. This interaction is proposed as a crucial step for B19V uptake and productive infection in permissive cells [21,22]. Thus, the presence of the indicated receptors was evaluated. Flow cytometry data show that α_5_β_1_ integrin was widely distributed on both BM- and FM-MSCs, and globoside was expressed in 50% of cells in both populations, suggesting that the virus may enter into some of these cells (Figure 3).

A truncated form of the VP1u protein was used to stain the VP1uR (Figure 3C). In contrast to the extended expression of the other receptors, the VP1uR was not detected in MSCs. UT7/EpoS1 cells that expressed the VP1uR were used as the control in the assay.

### 2.3. B19V Replication in FM-MSCs and BM-MSCs

**Figure 4:**
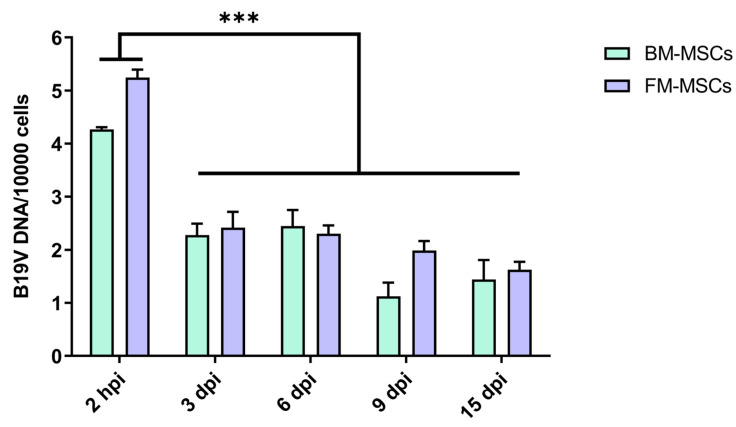
Amounts of B19V DNA in BM-MSCs and FM-MSCs. MSCs were infected with B19V at the multiplicity of 10^4^ geq/cell. The amount of B19V DNA was determined by qPCR at different times post-infection and expressed as B19V genome copies/10,000 cells. Columns indicate the mean values obtained from three independent experiments, bars are the standard error of means. Statistical analysis was performed by a Tukey’s multiple comparison test (***, *p* < 0.001).

We then assessed the permissiveness of MSCs to B19V. Cells were incubated with B19V at an MOI of 10^4^ geq/cell and then extensively washed to remove unbound virus. Infected cells were collected at different times post-infection to determine the amount of B19V DNA present within cells by qPCR (Figure 4). Viral DNA was quantified following absorption, 4.3 log and 5.2 log at 2 hpi in BM-MSCs and FM-MSCs, respectively, in spite of the extensive washes undertaken to remove the inoculum. Then, no evidence of viral replication was observed under our experimental conditions, since DNA decreased following 3 dpi and without further significant changes up to 15 dpi (3 log decrease). It is worth noting that the B19V DNA was still detected during the full course of infection, at the low but significant level of (copies/cell). This observation indicates that both BM-MSCs and FM-MSCs are susceptible to B19V infection, and constitute a non-permissive environment for achieving a productive replicative cycle, but may act as a reservoir for the persistence of B19V.

### 2.4. B19V Transcription in BM-MSCs and FM-MSCs

In vitro experimental studies suggest that limited genome expression may occur even on a non-replicating template. We thus investigated the viral transcription activity by qRT-PCR using different primer pairs. The contiguous primer pair 2210–2355 detected the whole set of viral transcripts, while the pair 1882–2033 detected the unspliced mRNA coding for the NS protein. Viral RNA was not detected at any time points analyzed, despite the intracellular persistence of viral DNA.

### 2.5. Immunophenotype of Infected MSCs

**Figure 5:**
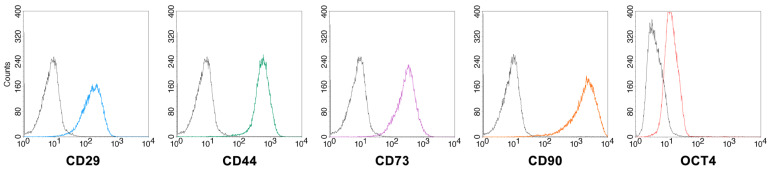
Immunophenotype of infected MSCs (BM-derived). Selected mesenchymal markers were analyzed by flow cytometry after 9 days of infection. Grey line: control; black line: specific antibody.

We next assessed whether the prolonged presence of B19V DNA affected the former identity of cells. The morphology of infected MSCs was similar to that of cells growing without the virus, and no cytopathic effect was visible during the course of infection. The expression of specific MSC markers was evaluated in infected cells at 9 days post infection, and compared to uninfected cells. The expression patterns of the selected markers showed no alteration in response to B19V exposure (Figure 5).

## 3. Discussion

In our study, we investigated the potential susceptibility and permissiveness to B19V infection of MSCs derived from two sources with different availability, which are significant in the context of B19V pathogenesis: human bone marrow and fetal membranes. MSCs have been demonstrated to be susceptible to infection by a variety of viruses representing prominent pathogens in immunocompromised hosts, including HSV-1, VZV, and CMV [24,25]. The pathogenic role of B19V in various MSC populations is poorly understood. B19V DNA was found in MSCs isolated from BM aspirates of healthy donors, and the corresponding B19V-positive MSCs were suggested to transmit the virus to bone marrow cells [26], while cultured synovium MSCs were shown to be unable to support viral replication [27]. Hence, whether MSCs of different sources are susceptible to B19V is still unclear.

MSCs may possess various surface receptors that can determine their susceptibility to viral infection, so we first assessed the expression of cellular receptors related to B19V tropism. Both BM- and FM-MSCs express globoside, which is thought of as the primary attachment receptor of B19V and required for infectivity, and integrin α_5_β_1_, one of the co-receptors proposed. Recently, the interaction of a cell receptor that specifically binds the B19V-VP1u region has also been proposed to be crucial to the productive infection in permissive cells. Using a truncated form of the B19V-VP1u protein, we thus evaluated the presence of this VP1u-receptor (VP1uR) via flow cytometry. VP1uR expression was not observed in both cellular types, although the detection of globoside and integrin α5β1 suggests that these MSCs populations might be susceptible to B19V infection. Indeed, B19V DNA was found within MSCs following viral absorption (4.3 log and 5.2 log at 2 hpi in BM-MSCs and FM-MSCs, respectively), confirming that viral entry/attachment can occur. Nevertheless, in our experimental conditions, we did not observe a productive infection of BM- and FM-MSCs. qPCR analysis showed that cells are not able to support viral replication, and the amount of B19V DNA progressively decreased during the course of infection, up to 15 dpi (3 log overall decrease). Permissiveness to B19V replication is known to be a multifactorial process influenced by interaction with specific receptors, erythropoietin pathway activation and host cellular factors. As a consequence, viral replication appears to be highly restricted in different cell types [28]. Abortive infections can be characterized by the production of low levels of viral transcripts without significant increases in B19V DNA levels. Differential expression of the distinct classes of viral transcripts has been observed in B19V permissive and nonpermissive cells. In nonpermissive cells, where a restricted transcriptional activity can occur, mRNAs encoding capsid proteins are limited, leading to the accumulation of NS transcripts [10]. We thus investigated the transcriptional activity in infected MSCs by qRT-PCR, using two different primer pairs for the amplification of total B19V RNA and specific mRNAs of the NS protein. Despite the B19V promoter being active in many cell types, viral RNA transcription was not allowed in BM- and FM-MSCs under these experimental conditions. Virus–cell interactions can induce functional changes in MSCs, affecting their expected differentiation and immunomodulatory properties [25]. Since B19V DNA was still detected at late time points in the infected cultures, we analyzed the maintenance of the expression pattern of mesenchymal markers in infected MSCs. Flow cytometry data show that the presence of B19V DNA did not alter the specific MSC markers’ expressions (such as CD29, CD44, CD73, CD90 and OCT4) after 9 days of infection compared to the phenotype of uninfected cells. However, we cannot exclude the possibility that different cellular conditions might alter the apparently quiescent state of B19V DNA in MSCs. Moreover, the non-permissive MSCs with a persistent presence of B19V DNA might act as a viral reservoir, increasing the risk of viral transmission, and for this reason, they can represent a greater risk, especially for immunocompromised patients. Further investigations should be performed to understand the role of B19V infection in MSCs, for example, in co-cultures with erythroid progenitor cells and in 3D cell cultures, as a more relevant model of in vivo conditions [29,30,31]. It has to be remembered that the clinical efficacy of MSCs involves the cellular administration to a recipient subject, where they can contribute to healing [32]. In this context, the potential reactivation of B19V should be properly studied, as well as the experimental culture conditions, prior to the cell transplantation, which could change the susceptibility of the in vitro manipulated MSCs to the virus itself. In addition, MSCs play a crucial role in the maintenance of the hematopoietic stem cell niche in the bone marrow; whether and how BM-resident stromal cells targeted by B19V will be affected on their hematopoietic supportive capacity still remains an open question. A second aspect of interest is the vertical transmission of B19V. As far as we know, this is the first time that FM-MSCs have been infected with B19V in vitro. Although FM-MSCs were found to be non-permissive to B19V infection, studying virus–cell interactions at the maternal interface will provide more information about B19V pathogenesis during pregnancy. Alternatively, by studying and comparing the MSCs with similar properties but with different tissue localizations could help us to understand the essential mechanisms at the basis of the susceptibility and permissiveness of MSCs to B19V.

## 4. Materials and Methods

### 4.1. MSCs Isolation and Culture

BM-MSCs were obtained from bone marrow aspirates of healthy adult volunteers. Samples were diluted 1:3 with PBS and layered over a Ficoll–Histopaque gradient (1.077 g/mL; Sigma, St. Louis, MO, USA). The isolated mononuclear cells were seeded at 10^6^/cm^2^ in Dulbecco’s modified eagle’s medium (DMEM, Sigma) supplemented with 20% heat-inactivated fetal bovine serum (FBS, Sigma), 200 U/mL penicillin and 200 μg/mL streptomycin, at 37 °C in 5% CO_2_.

FM-MSCs were isolated from the term placenta of healthy donor mothers obtained after written informed consent during cesarean section. This study was approved by the Local Ethical Committee (IRCCS St. Orsola-Malpighi University Hospital Ethical Committee, protocol n° 2481/2017, ref n° 68/2017/U/Tess). Fetal membrane samples were prepared in accordance with a previously established protocol [7], and cells were finally seeded in DMEM with 20% FBS, at 37 °C in 5% CO_2_. Cells were assessed for suitable osteogenic and adipogenic differentiation, as previously described [33].

For both cultures, non-adherent cells were removed after one week, and subcultures were maintained in DMEM supplemented with 10% FBS.

### 4.2. Flow Cytometry

Cells were characterized by flow cytometry for the expression of specific antigens defining the identity of MSCs, as well as for B19V receptor moieties. Briefly, trypsinized cells were stained for 30 min at 4 °C with the following monoclonal antibodies: anti-CD29-FITC, anti-CD44-FITC, anti-CD73-PE and anti-CD90-PC5 (all from Beckman Coulter, Fullerton, CA, USA). For the evaluation of Octamer binding transcription factor (OCT4) expression, cells were first permeabilized using the IntraPrep Permeabilization Reagent (Beckman Coulter), then incubated with the mouse monoclonal antibody anti-OCT3/4 (Santa Cruz Biotechnology Inc., Santa Cruz, CA, USA), and subsequently with the anti-mouse-Alexa Fluor 488 antibody (Invitrogen, Thermo Fisher Scientific, Waltham, MA, USA), performing each step at room temperature for 30 min.

Globoside and α_5_β_1_ integrin expression was evaluated using polyclonal rabbit anti-globoside (Matreya, State College, PA, USA) and monoclonal mouse anti-α_5_β_1_ (Immunological Sciences, Rome, Italy) antibody, followed by anti-rabbit-FITC (Dako, Glostrup, Denmark) and anti-mouse-Alexa Fluor 488 (Invitrogen) antibody, respectively; each incubation was performed at 4 °C for 30 min.

For VP1uR, trypsinized cells were blocked in PBS/10% FCS for 20 min at 4 °C, then incubated for 1 h at 4 °C with the complex ΔC128 protein/anti-FLAG antibody (Agilent Technologies, Cedar Creek, TX, USA), previously prepared in PBS/2% FCS at a ratio of 75 ng/300 ng. ΔC128 is a truncated form of the B19V-VP1u protein, corresponding to the first 100 aa, which contain the receptor-binding domain (RBD) and a FLAG tag for detection (a kind gift of Dr. Carlos Ros, University of Bern, Bern, Switzerland [23]). The anti-rat-DyLight488 antibody (ImmunoReagents Inc., Raleigh, NC, USA) was used as a secondary antibody for 1 h at 4 °C.

Labeled cells were washed twice in PBS before being analyzed with an FACSCalibur cytometer using the Cell Quest Pro Software Version 1.0 (Becton Dickinson, East Rutherford, NJ, USA).

### 4.3. Virus and Infection

A stock of B19V was generated in vitro, starting from a cloned synthetic construct corresponding to the complete B19V genome, as previously described [34,35]. For infection experiments, trypsinized cells were resuspended in PBS at a density of 10^7^ cells/mL and infected with stock virus at a multiplicity of infection (MOI) of 10^4^ genome copies (geq)/cell. After the absorption period of 2 h at 37 °C, infected cells were washed and seeded with fresh medium at an initial density of 10^4^ cell/cm^2^.

### 4.4. Nucleic Acids Purification

Cell cultures were collected at different times following infection and processed using the Maxwell Viral Total Nucleic Acid Purification Kit on a Maxwell Instrument (Promega, Fitchburg, WI, USA), in order to obtain a total nucleic acids fraction in elution volumes of 120 µL. For DNA analysis, an aliquot of the eluted nucleic acids was directly amplified, while for RNA amplification, aliquots of experimental samples were treated with Turbo DNAfree reagent (Ambion, Thermo Fisher Scientific, Waltham, MA, USA) before the amplification step.

### 4.5. qPCR and qRT-PCR for Detection of B19V Nucleic Acids

Quantitative real-time PCR (qPCR) and RT-PCR (qRT-PCR) were performed using the QuantiTect SybrGreen PCR Kit and QuantiTect SybrGreen RT-PCR Kit on a RotorQ system (Qiagen, Hilden, Germany), including 0.5 µM of each specific primer pair, according to previously established protocols [10]. Quantitation of viral nucleic acids was obtained by the absolute quantitation algorithm, converting quantification cycle (Cq) values to geq number using external calibration curves obtained from respective standard targets. The primers used for the amplification of B19V DNA and the whole set of viral transcripts were the pair R2210–R2355, located in the central exon of the B19V genome, while determination of NS transcripts was obtained by using the pair R1882–R2033. For control and normalization with respect to the number of cells, a target sequence in the region of genomic DNA coding for 18S rRNA (rDNA) was amplified. All primers used are listed in Table 1.

### 4.6. Statistical Analysis

Statistical analysis was performed using GraphPad Prism software (9.5 for Windows). Data obtained from three independent experiments have been calculated as mean ± SD and analyzed using ANOVA test. Differences were considered statistically significant when *p* ≤ 0.0001.

## Figures and Tables

**Table 1:** Primers used in qPCR and qRT-PCR assays.

Primer	Sense	Primer	Antisense	Target
18Sfor	CGGACAGGATTGACAGATTG	18Srev	TGCCAGAGTCTCGTTCGTTA	Genomic 18S rDNA
R2210	CGCCTGGAACACTGAAACCC	R2355	GAAACTGGTCTGCCAAAGGT	Virus DNA/total RNA
R1882	GCGGGAACACTACAACAACT	R2033	GTCCCAGCTTTGTGCATTAC	NS mRNA

## Data Availability

Not applicable.
